# Inflammatory Markers in Combination With Multiple Patterns of Extrarenal Extension Accurately Indicate Recurrence Risk in Patients With pT3aN0M0 Clear Cell Renal Cell Carcinoma

**DOI:** 10.1002/cnr2.70371

**Published:** 2025-10-18

**Authors:** Masato Uetani, Fumito Yamabe, Shunsuke Hori, Mizuki Kasahara, Mizuho Okawa, Hideyuki Kobayashi, Koichi Nagao, Koichi Nakajima, Yozo Mitsui

**Affiliations:** ^1^ Department of Urology Toho University Faculty of Medicine Tokyo Japan

**Keywords:** inflammatory markers, kidney cancer, pembrolizumab, recurrence, tumor staging

## Abstract

**Background and Aims:**

A clear understanding of the risk of clear cell renal cell carcinoma (ccRCC) recurrence in individual patients is essential for appropriate administration of adjuvant therapy. Notably, non‐metastatic pT3a ccRCCs are heterogeneous, and affected patients will have a different prognosis depending on the pathological definition. The present study was conducted to identify factors associated with postoperative recurrence in pT3a ccRCC cases.

**Methods:**

We retrospectively reviewed 295 patients who underwent a radical or partial nephrectomy for RCC at our institution from 2013 to 2022 and identified 42 with pT3aN0M0 ccRCC. Preoperative clinical and pathological data, including blood parameters, were collected, and their association with recurrence‐free survival (RFS) was evaluated. The Kaplan–Meier method and uni‐ and multivariable Cox proportional hazard regression were used for statistical analysis.

**Results:**

Univariate and subsequent multivariate analyses showed white blood cells, platelets, and mixed patterns of pT3a components as independent prognostic factors with significant relationships with RFS. Kaplan–Meier curves revealed that the presence of leukocytosis or thrombocytosis was associated with worse RFS (*p* = 0.0002, *p* < 0.0001, respectively), while patients with a mixed pattern of pT3a components also had worse RFS as compared to those with a single pattern (*p* = 0.0007). Furthermore, accuracy for prediction of RFS in pT3a ccRCC cases was improved by classification based on the number of retained factors, that is, leukocytosis, thrombocytosis, and mixed patterns of pT3a components.

**Conclusions:**

The present findings indicate that inflammatory markers, such as leukocytes and platelets, as well as multiple patterns of extrarenal extension are factors useful for predicting RFS in patients with pT3aN0M0 ccRCC. Their combined use should aid in the selection of appropriate postoperative adjuvant therapy.

## 1 Introduction

Renal cell carcinoma (RCC) is the third most common urological cancer, accounting for 3% of all cases, with approximately 70% of these reported to be clear cell RCC (ccRCC) [1, 2]. The gold standard treatment for nonmetastatic RCC is a radical or partial nephrectomy procedure, with various methods available, including open, laparoscopic, and robot‐assisted approaches. Unfortunately, even after undergoing complete surgical resection, approximately 30% of affected patients develop metastasis or recurrence, and the 5‐year survival rate for such cases is poor at less than 10% [3, 4]. However, sufficient evidence regarding the effectiveness of perioperative treatment has not been shown, partly because no method for the evaluation of recurrence risk in postoperative RCC cases has been fully established.

Recent findings obtained in the KEYNOTE‐564 trial demonstrated the efficacy of pembrolizumab as postoperative adjuvant treatment for ccRCC, which became available in Japan in August 2022 for ccRCC patients with a high risk of recurrence [5, 6]. For that trial, risk groups were established to provide a clear definition of high recurrence, as follows: intermediate‐high risk (pT2 with grade 4 or N0 and M0 with sarcomatoid change, pT3 with N0 and M0 regardless of grade), high risk (pT4 with N0 and M0 regardless of grade, N1 and M0 regardless of pT or grade), and M1 NED (no evidence of disease). According to the definition used in the KEYNOTE‐564 trial, pT3 ccRCC patients are uniformly considered to have a high risk of recurrence. However, previous studies have shown that the recurrence rate of pT3a cases is significantly lower compared to that of pT3b‐c and nearly the same as that of pT2 cases [7]. Furthermore, pT3a constitutes the majority of pT3 cases and, while it has been reported that pT3 patients overall have a shorter time to recurrence compared to pT2, the recurrence rate may not be significantly different between those two groups [7, 8]. Therefore, to assist with the appropriate selection of pembrolizumab as adjuvant therapy for pT3 ccRCC, the risk of recurrence for pT3a should be considered on an individual basis.

The 8th edition of the TNM system classifies the pT3a stage of RCC as tumors with perirenal fat infiltration (PFI), renal sinus fat invasion (SFI), or renal vein invasion (RVI) [9]. Thus, each case has a different invasion pattern, and several studies have shown that this pathological heterogeneity may influence differences in oncological outcomes of pT3a RCC patients [10, 11, 12, 13]. In addition, other pathological factors, such as tumor grade, size, and necrotic tissue, may also have effects on recurrence and prognosis of pT3a RCC [12, 13, 14, 15]. Regarding host factors, relationships of preoperative blood parameters, such as anemia and elevated inflammation‐related markers including platelets and fibrinogen, with poor oncological outcomes after surgery have also been shown [16, 17]. As a result, a variety of factors associated with RCC may be individually or complementarily related to prognosis. The present study was conducted to identify clinical and pathological factors that more accurately predict postoperative recurrence in pT3aN0M0 ccRCC cases.

## 2 Materials and Methods

### 2.1 Patient Population

**FIGURE 1:**
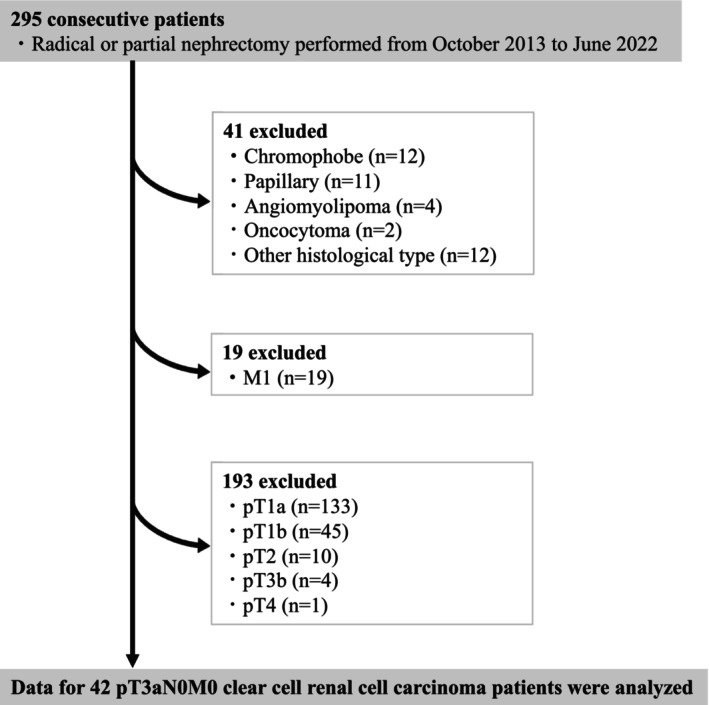
Inclusion and exclusion criteria.

The records of 295 patients with a renal tumor who underwent a radical or partial nephrectomy procedure at our institution between October 1, 2013 and June 30, 2022 were retrospectively reviewed. A flow chart showing patient inclusion and exclusion criteria is presented in Figure 1. Parameters used for exclusion criteria were obtained sequentially. Patients included in this study were determined based on the following parameters. First, 41 patients with a histological type other than ccRCC and 19 with distant metastasis were excluded. Subsequently, tumors in the remaining cases were restaged according to the 2017 TNM system [9] and 193 patients with a disease stage other than pT3a were excluded. Thus, findings obtained for 42 pT3aN0M0 ccRCC patients were used for further analysis. None of the selected pT3a patients received postoperative adjuvant therapy, such as interferon or interleukin 2 treatment. Additionally, data from 188 patients with pT1‐2 ccRCC were used to explore the significance of inflammatory markers in low‐stage cases. Due to the retrospective nature of this study, the process of obtaining patient consent was omitted. Instead, information about the study was disclosed on the website of the hospital and potential participants were given an opportunity to opt out. This study was conducted following approval from the Toho University Omori Medical Center Ethics Committee (number M23098) in accordance with the Declaration of Helsinki.

### 2.2 Assessments

Clinicopathological characteristics, including age, body mass index (BMI), Eastern Cooperative Oncology Group status (ECOG PS), gender, comorbidities such as hypertension and diabetes mellitus, blood parameters such as serum hemoglobin (Hgb), white blood cell (WBC), platelet (PLT), lactate dehydrogenase (LDH), alkaline phosphatase (ALP), total protein (TP), albumin (Alb), and C‐reactive protein (CRP) levels, clinical tumor T stage, tumor size, tumor pathology (nuclear Fuhrman grade, vascular and lymphatic invasion, and necrosis), tumor invasion status (PFI, SFI, RVI, and combinations), and surgical technique (radical or partial) were collected and assessed. Blood parameters used were obtained from preoperative blood collections routinely performed within 2 weeks before surgery. When subjective symptoms such as fever are present, surgery is generally postponed. Therefore, it was considered that the values obtained for inflammatory markers were not affected by infection. Based on standard values used at our hospital, Hgb < 12.5 g/dL (regardless of age and gender), LDH ≥ 220 U/L, ALP ≥ 260 U/L, TP < 8.0 g/dL, Alb < 3.5 g/dL, and CRP ≥ 0.2 mg/dL were defined as abnormal. Therefore, in this study, WBC ≥ 9000/μL and PLT ≥ 360 000 μL were considered to be leukocytosis and thrombocytosis, respectively. Age and BMI were each divided into two groups based on the median values; ECOG PS was divided based on 0 or 1 or more; Fuhrman grade was divided based on 2 or less or 3 or more; and tumor diameter was divided based on less than or equal to or greater than 7 cm [14]. Clinical stage was diagnosed based on preoperative chest and abdominal computerized tomography (CT) and chest X‐rays, with abdominal magnetic resonance imaging results referred to when necessary. Postoperatively, chest and abdominal CT scanning was performed every 3–6 months for the first 5 years and annually thereafter. The primary endpoint of the study was recurrence‐free survival (RFS), which was defined as the time from surgery to initial recurrence shown by imaging.

### 2.3 Statistical Analyses

Measurement values are shown as median (interquartile range; IQR) or number (percent of total). RFS curves were created using the Kaplan–Meier method and differences between them were analyzed with a log‐rank test. Uni‐ and multivariate Cox proportional hazard models were employed to determine risk factors for RFS. *p* values less than 0.05 were considered to indicate statistical significance. All data were analyzed using the EZR statistical software application (R, version 4.3.1, 2023) (http://www.jichi.ac.jp/saitama‐sct/SaitamaHP.files/statmed.html) [18].

## 3 Results

### 3.1 Patient Characteristics

The clinicopathological characteristics of the 42 pT3aN0M0 ccRCC patients included in this study are summarized in Table 1. Median age at the time of surgery for RCC was 70.0 years (60.8–78.3 years) and BMI was 23.0 kg/m^2^ (22.0–25.3 kg/m^2^). Twenty‐two (52.4%) patients had a preoperative PS score of 0, while the remaining 20 (47.6%) had a score of 1 or higher. Of the 42 patients, nine (21.4%) were women, while 30 (71.4%) had hypertension, and 11 (26.2%) had diabetes. Among the preoperative blood markers noted, the median level of Hgb was 13.3 g/dL (12.5–14.4 g/dL), of WBC was 6800/μL (5600–8000/μL), of PLT was 249 000 μL (202 000–287 000 μL), of TP was 7.7 g/dL (7.3–8.0 g/dL), and of CRP was 0.2 mg/L (0.1–0.9 mg/L). As for patterns of extrarenal tumor extension status, PFI, SFI, and RVI were observed in 25 (59.5%), 13 (31.0%), and 15 (35.7%) cases, respectively, while more than one of those were noted in 10 (23.8%). The median follow‐up period was 37 (17.8–59.8) months, with eight (19.0%) patients showing recurrence and one (2.4%) death because of ccRCC during the observation period.

**TABLE 1:** Clinicopathological characteristics of 42 patients with pT3a clear cell renal cell carcinoma.

Characteristics	*N* = 42
Age at surgery, years	70.0 (60.8–78.3)
Body mass index, kg/m^2^	23.0 (22.0–25.3)
ECOG PS	
0	22 (52.4)
≥ 1	20 (47.6)
Gender	
Female	9 (21.4)
Male	33 (78.6)
Comorbidities	
Hypertension	30 (71.4)
Diabetes mellitus	11 (26.2)
Serum markers before surgery	
Hemoglobin, g/dL	13.3 (12.5–14.4)
White blood cells, /μL	6800 (5600–8000)
Platelet, μL	249 000 (202 000‐287 000)
Lactate dehydrogenase, U/L	206 (183–230)
Alkaline phosphatase, U/L	215 (144–276)
Total protein, g/dL	7.7 (7.3–8.0)
Albumin, g/dL	4.1 (3.7–4.2)
CRP, mg/L	0.2 (0.1–0.9)
Clinical T stage	
≤ T2	32 (76.2)
≥ T3	10 (23.8)
Tumor diameter, cm	5.8 (3.7–7.0)
Fuhrman grade	
≤ 2	37 (88.1)
≥ 3	5 (11.9)
Vascular invasion	32 (76.2)
Lymphatic invasion	8 (19.5)
Tumor necrosis	8 (19.5)
pT3a components	
Perinephric fat invasion	25 (59.5)
Sinus fat invasion	13 (31.0)
Renal vein invasion	15 (35.7)
Any two or more	10 (23.8)
Surgical technique	
Radical nephrectomy	34 (81.0)
Partial nephrectomy	8 (19.5)

*Note:* Data are presented as median (interquartile range), or number (percentage).

Abbreviations: CRP, c‐reacted protein; ECOG PS, Eastern Cooperative Oncology Group Performance Status scale.

### 3.2 Postoperative Recurrence Predictors

Univariate Cox proportional hazard regression was initially performed using several factors, including blood and clinicopathological parameters, to determine candidate factors associated with postoperative recurrence. As shown in Table 2, among the hematologic parameters, increased WBC and PLT levels were independent variables significantly associated with postoperative recurrence (*p* = 0.0028 and *p* = 0.0051, respectively). Furthermore, CRP level, another representative serum inflammatory marker, also showed a tendency for correlation with RFS but did not reach statistical significance (*p* = 0.0710). Regarding histopathological factors, tumor diameter (> 7 cm), tumor grade, and necrosis, considered to be candidate prognostic factors in previous studies [11, 12, 13, 14, 15], were not significantly associated with postoperative recurrence. In addition, when PFI, SFI, or RVI, factors associated with extrarenal tumor extension status and the basis for diagnosis of pT3a, were noted alone, they were not significantly associated with postoperative recurrence, whereas those in various combinations were found to be significantly associated with RFS in the present pT3a ccRCC cases. Increased WBC and PLT levels and a mixed pT3a component pattern were significant candidate predictors of RFS in univariate analysis, with each also identified as an independent predictor of RFS in subsequent multivariate analysis findings (Table 2).

**TABLE 2:** Uni‐ and multivariate Cox proportional hazards analysis findings for cancer recurrence after surgery.

Covariates	Univariate		Multivariate	
	HR (95% CI)	*p*	HR (95% CI)	*p*
Age at surgery (< 70 vs. ≥ 70 years)	1.911 (0.409–8.937)	0.4105	—	—
Body mass index (< 23 vs. ≥ 23 kg/m^2^)	0.631 (0.152–2.614)	0.5256	—	—
ECOG PS (0 vs. ≥ 1)	0.971 (0.226–4.175)	0.9687	—	—
Gender (female vs. male)	2.093 (0.380–11.52)	0.3958	—	—
Hypertension (absent vs. present)	2.664 (0.617–11.51)	0.1894	—	—
Diabetes mellitus (absent vs. present)	2.072 (0.254–16.94)	0.4966	—	—
Hemoglobin (< 12.5 vs. ≥ 12.5 g/dL)	0.404 (0.090–1.810)	0.2360	—	—
White blood cells (< 9000/μL vs. ≥ 9000/μL)	11.91 (2.350–60.33)	0.0028	48.48 (2.715–865.7)	0.0083
Platelet (< 360 000 vs. ≥ 360 000 μL)	23.53 (2.581–214.5)	0.0051	20.70 (1.292–331.6)	0.0322
Lactate dehydrogenase (< 220 vs. ≥ 220 U/L)	1.483 (0.352–6.259)	0.5913	—	—
Alkaline phosphatase (< 260 vs. ≥ 260 U/L)	3.357 (0.745–15.12)	0.1147	—	—
Total protein (< 8.0 vs. ≥ 8.0 g/dL)	2.132 (0.473–9.605)	0.3243	—	—
Albumin (< 3.5 vs. ≥ 3.5 g/dL)	0.214 (0.043–1.070)	0.6040	—	—
CRP (< 0.2 vs. ≥ 0.2 mg/dL)	6.924 (0.847–6.924)	0.0710	—	—
Tumor diameter (< 7 vs. ≥ 7 cm)	5.132 (1.322–28.31)	0.0605	—	—
Fuhrman grade (≤ 2 vs. ≥ 3)	1.032 (0.930–8.818)	0.9773	—	—
Vascular invasion (absent vs. present)	1.702 (0.199–14.59)	0.6274	—	—
Lymphatic invasion (absent vs. present)	0.699 (0.081–6.018)	0.7448	—	—
Tumor necrosis (absent vs. present)	3.890 (0.601–25.18)	0.1539	—	—
pT3a component, perinephric fat invasion (absent vs. present)	4.011 (0.752–21.39)	0.1039	—	—
pT3a component, sinus fat invasion (absent vs. present)	2.430 (0.603–9.789)	0.2116	—	—
pT3a component, renal vein invasion (absent vs. present)	1.621 (0.363–7.228)	0.5267	—	—
pT3a component (any two or more vs. one)	9.854 (1.967–49.37)	0.0054	10.684 (1.166–97.86)	0.0360
Surgical technique (radical vs. partial)	0.899 (0.105–7.698)	0.9223	—	—

Abbreviations: CRP, C‐reactive protein; ECOG PS, Eastern Cooperative Oncology Group Performance Status Scale; HR, hazard ratio.

### 3.3 Oncological Outcome Based on Presence of Leukocytosis, Thrombocytosis, and Mixed pT3a Component Pattern

**FIGURE 2:**
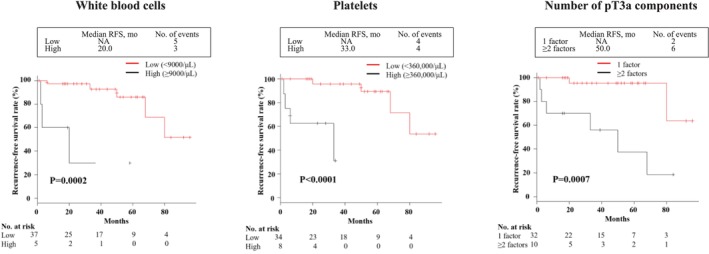
Kaplan–Meier analysis of recurrence‐free survival after surgical treatment for pT3a clear cell renal cell carcinoma according to numbers of white blood cells, platelets, and pT3a components. As compared to the normal group, recurrence‐free survival (RFS) was significantly shorter in the leukocytosis (*p* = 0.0002) and thrombocytosis (*p* < 0.0001) groups. Furthermore, the group with two or more pT3a components had significantly worse RFS as compared to any of the groups with only one component (*p* = 0.0007).

**FIGURE 3:**
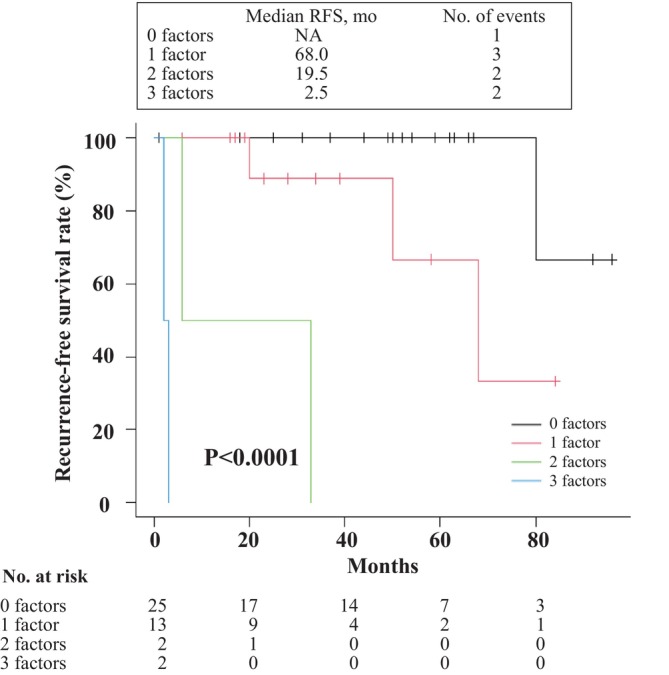
Kaplan–Meier analysis of recurrence‐free survival after surgical treatment for pT3a clear cell renal cell carcinoma based on combining numbers of pT3a components and inflammatory markers. Patients without multiple extrarenal infiltrative patterns or either of the two inflammatory markers showed the best RFS, while that decreased significantly with increasing numbers of these factors (*p* < 0.0001).

Next, RFS curves for each of the three candidate predictors identified by Cox regression findings were calculated using Kaplan–Meier survival analysis, as shown in Figure 2. RFS was significantly shorter in patients with leukocytosis (≥ 9000/μL) as compared to those with a normal WBC level (median RFS: 19.0 vs. 37.0 months, *p* = 0.0002). For reference, RFS stratification for the four groups based on WBC value is also shown in Figure S1. Similarly, RFS for the thrombocytosis group (≥ 360 000 μL) was significantly shorter than that for the normal group (median RFS: 14.5 vs. 44.0 months, *p* < 0.0001). In addition, patients with two or more of PFI, SFI, and RVI had significantly worse RFS than those with any one of these factors alone (median RFS: 25.0 vs. 35.5 months, *p* = 0.0007). It was also noted that further stratification based on combinations of multiple extrarenal infiltration patterns and elevated serum WBC and PLT levels identified a stepwise reduction in time to recurrence in patients with pT3a cRCC, which was dependent on the number of these factors noted (Figure 3).

**FIGURE 4:**
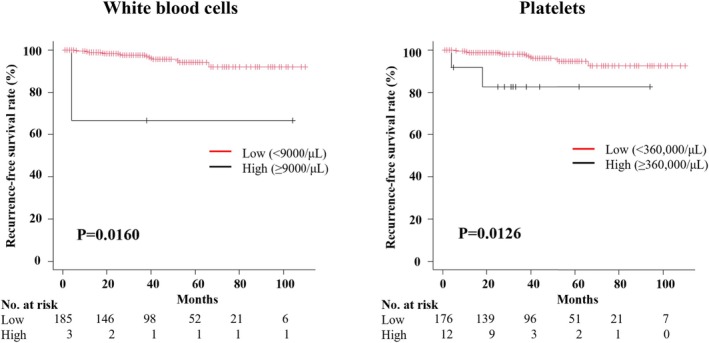
Kaplan–Meier analysis of recurrence‐free survival after surgical treatment for pT1–2 clear cell renal cell carcinoma. Patients with T1‐T2 clear cell renal cell carcinoma, along with a high white blood cell or platelet count had significantly worse recurrence‐free survival than patients with normal white blood cell and platelet counts.

Finally, the prognostic significance of the inflammatory markers identified in this study for lower stage ccRCC was examined. This evaluation included 188 nonmetastatic T1–T2 ccRCC cases, of which nine (4.8%) developed tumor recurrence during the follow‐up period. The median WBC and PLT levels for all pT1–T2 cases were 5900/μL (5000–7175/μL) and 225 500 μL (185 500–273 000 μL), respectively, both lower than those for all pT3a patients. Details of these 188 T1–T2 cases are shown in Table S1. Notably, even among T1–T2 cases, those with a high white blood cell or platelet count were found to have a significantly worse prognosis (Figure 4).

## 4 Discussion

Pembrolizumab has emerged as a new treatment option in the recently established field of postoperative adjuvant therapy for ccRCC. Even without lymph node or distant metastasis, pembrolizumab is indicated for cases classified as pT3 or higher according to the eligibility criteria used for the KEYNOTE‐564 trial [5, 6]. However, in consideration of the heterogeneous prognosis findings of affected patients, the effectiveness of a uniform postoperative adjuvant therapy for pT3a cases is debatable. Findings obtained in the present study indicate that preoperative elevated levels of peripheral blood WBCs and PLTs, as well as multiple patterns of pT3a components (two or more of PFI, SFI, and RVI present) are findings useful for the prediction of postoperative recurrence. Furthermore, more accurate prediction of RFS was obtained when these inflammatory markers, suggested to have a role in regard to the prognosis of localized RCC in previous studies [16, 17, 19], were used in combination with multiple patterns of pT3a components. Therefore, with the use of these three factors, it is possible to select individuals with a poor prognosis from a heterogeneous group of pT3a patients. Although several previous studies have focused on relationships between pT3a component patterns and prognosis, the present study is the first to present findings obtained with combinations of inflammatory markers. It is considered that these results can be useful for determining the need for adjuvant therapy as well as the selection of pT3aN0M0 ccRCC patients most likely to benefit from such treatment.

There is currently no consensus regarding differences in prognostic significance among the invasion patterns PFI, SFI, and RVI, which are utilized as diagnostic criteria for pT3a RCC. Bedke et al. [20] reported that the prognosis of pT3a RCC cases with SFI was significantly worse as compared to those with PFI. A meta‐analysis performed by Zhang et al. [10] also found that SFI was associated with worse prognosis for pT3a RCC patients. Additionally, it has been reported that only PFI was found to correlate with poor prognosis in pT3a RCC patients [13]. On the other hand, there are also studies that found no difference in prognostic significance among these three factors [11, 12, 14]. In the present investigation as well, there was no significant effect on postoperative recurrence found due to differences among the three infiltration patterns. However, it is important to note that patients with two or more infiltration patterns had significantly shorter RFS as compared to those with only one. This finding is in line with previous reports showing that pT3a RCC patients with any two or more of PFI, SFI, and RVI had worse prognosis [11, 12, 20, 21]. Although the full impact of each of these three invasion patterns on prognosis and recurrence remains unclear, results obtained in the present as well as previous studies suggest that the presence of multiple patterns is associated with higher rates of disease progression and cancer‐related mortality after surgery as compared with only a single pattern.

Inflammation is generally believed to influence each step of tumor development, from tumorigenesis to metastasis [22, 23]. Therefore, it is not surprising that increased systemic inflammatory markers may be significantly associated with prognosis associated with various types of cancer. Indeed, systemic inflammatory markers, such as CRP, WBC, and Alb, as well as some indicators with relationships to these factors including fibrinogen‐albumin, have been reported to be applicable for determining the prognosis of RCC patients and the therapeutic efficacy of anti‐cancer drugs [16, 24]. In the present study, elevated CRP levels and leukocytosis, which indicate systemic inflammation, were included as candidate factors associated with postoperative recurrence, with leukocytosis identified as one of three independent factors useful for predicting recurrence.

Thrombocytosis is also thought to reflect systemic inflammation, as PLT can increase due to the presence of infection and/or inflammatory disease [25]. Interestingly, it has been shown that thrombocytosis in cancer patients is associated with decreased survival [26]. The present findings indicate that thrombocytosis is another blood biomarker useful for the prediction of postoperative recurrence in pT3aN0M0 ccRCC patients. Similarly, Xiao et al. [17] found that thrombocytosis in RCC cases was associated with an increased risk of early recurrence or metastasis. Another recent study showed that in addition to limiting blood loss and promoting wound healing, PLT plays a functional role in all stages of tumorigenesis, including tumor growth, tumor cell extravasation, and metastasis, by infiltrating the tumor microenvironment and directly interacting with cancer cells [26]. Also, it is noteworthy that the detection of tumor‐derived PLTs was found to hold promise as a minimally invasive blood biomarker for the diagnosis of RCC, as tumor cells alter the PLT RNA profile [27], though tumor cell‐derived PLTs were not evaluated in the present study.

Thrombocytosis is well known as one of the components of the International Metastatic RCC Database Consortium (IMDC) risk classification, which provides important information regarding prognosis and treatment response for patients with metastatic RCC [28]. Furthermore, an increase in neutrophils, a type of granulocyte in WBC, is one of the factors included in the IMDC risk classification. Notably, the present findings suggest that two inflammatory markers already established as poor prognostic factors in metastatic disease may also be applicable as factors related to recurrence of non‐metastatic ccRCC. Additionally, the present findings are the first to show that the addition of these two inflammatory markers to the possession of multiple invasion patterns, which has been reported as a candidate prognostic factor [11, 12, 20, 21], can lead to refinement of postoperative recurrence prediction in patients with pT3a ccRCC. It was also confirmed that an increase in these two inflammatory markers is proportional to an increase in stage and may also be applicable for predicting the prognosis of non‐metastatic T1–T2 ccRCC cases, though additional investigation is needed.

Apart from the pT3a component, several previous studies have presented candidate histopathological factors that may affect recurrence and prognosis of patients with pT3a ccRCC. Soltani et al. [12] showed that advanced nuclear Fuhrman grade and sarcomatoid change can be independent predictors of mortality in stage pT3a RCC patients. Furthermore, a large‐scale retrospective study conducted among 12 European centers with more than 1200 cases of pT3a RCC revealed that tumor size ≥ 7 cm was significantly correlated with poor prognosis [14] and also noted that the prognostic impact of the pT3a components PFI and RVI was comparable. Ohsugi et al. [13] reported that in addition to sarcomatoid changes, the presence of necrosis may be significantly associated with poor prognosis for patients with pT3a RCC. However, neither advanced nuclear Fuhrman grade nor tumor size ≥ 7 cm was found to be a significant predictor of poor prognosis in those cases. Although larger tumor size showed a tendency to be associated with a higher risk of recurrence, the present results indicate that none of these histopathological factors are significant predictors of RFS. It is possible that this discrepancy with other studies is due to the diversity of subject group numbers, treatment durations, patient ethnic origins, and treatment methods among investigations thus far conducted. Notably, there are differences for each histopathological factor and its role in predicting prognosis among studies presented thus far, and it is important to emphasize that the present results do not negate the importance of other findings.

This study has some limitations, including the retrospective design, data from a low number of patients treated at a single institution, and a short follow‐up period. These factors may have had an influence on the inability to reconfirm the prognostic significance of previously reported histopathological risk factors, such as tumor size, nuclear grade, and necrosis, as such findings vary among prior studies, which are noted above. Also, no external validation was conducted to support the reliability of the present findings using new data representative of the target population because the purpose of this study was to develop a prediction model. For the analyses, WBC and PLT values were classified into two groups based on the reference values used at our hospital, though more appropriate cut‐off values should also be considered. Additional studies that take these limitations into account will be needed to confirm the present results.

## 5 Conclusion

Findings obtained in the present multifaceted evaluation of postoperative risk of recurrence in pT3aN0M0 ccRCC patients using various factors, such as blood markers and pathological factors, indicate that inflammatory markers such as leukocytes and platelets, as well as multiple patterns of extrarenal invasion may predict RFS in pT3aN0M0 ccRCC patients. Although several candidate factors related to the prognosis of patients with pT3aN0M0 ccRCC have been proposed, including multiple patterns of extrarenal invasion, also identified in the present findings, this study is the first to find evidence supporting the significance of inflammatory markers. Additionally, it was demonstrated that combining two preoperative inflammatory markers into multiple patterns of extrarenal extension allows for more accurate prediction of postoperative recurrence in pT3aN0 cases. Although additional analyses are needed, it is considered that these results will be helpful for selection of pT3aN0M0 ccRCC patients who will benefit most from adjuvant therapy.

## Author Contributions

**Masato Uetani:** methodology. **Fumito Yamabe:** validation. **Shunsuke Hori:** data curation. **Mizuki Kasahara:** data curation. **Mizuho Okawa:** data curation. **Hideyuki Kobayashi:** formal analysis. **Koichi Nagao:** supervision. **Koichi Nakajima:** supervision. **Yozo Mitsui:** writing – original draft, methodology, conceptualization. All authors had full access to the data in the study and take responsibility for its integrity and the accuracy of data analysis.

## Ethics Statement

This study was conducted following approval from the Toho University Omori Medical Center Ethics Committee (no. M23098) in accordance with the Declaration of Helsinki.

## Conflicts of Interest

The authors declare no conflicts of interest.

## Supporting information


**Figure S1:** Kaplan–Meier analysis of recurrence‐free survival after surgical treatment for pT3a clear cell renal cell carcinoma in patients divided in four groups according to number of white blood cells. Subdivision into four groups based on white blood cell count revealed a stepwise decrease in recurrence curve associated with increased white blood cells.

**Table S1:** Clinicopathological characteristics of 188 patients with pT1‐2 clear cell renal cell carcinoma.

## Data Availability

The data that support the findings of this study are available from the corresponding author upon reasonable request.
