# Supplementary material for: Inflammatory Markers in Combination With Multiple Patterns of Extrarenal Extension Accurately Indicate Recurrence Risk in Patients With pT3aN0M0 Clear Cell Renal Cell Carcinoma
Source: Cancer Rep (Hoboken). 2025 Oct 18;8(10):e70371. doi: 10.1002/cnr2.70371 (PMC12535211; doi:10.1002/cnr2.70371)
Supplement: Supplementary file 1 — Figure S1: Kaplan–Meier analysis of recurrence‐free survival after surgical treatment for pT3a clear cell renal cell carcinoma in patients divided in four groups according to number of white blood cells. Subdivision into four groups based on white blood cell count revealed a stepwise decrease in recurrence curve associated with increased white blood cells. [file CNR2-8-e70371-s001.pptx]

## Slide 1
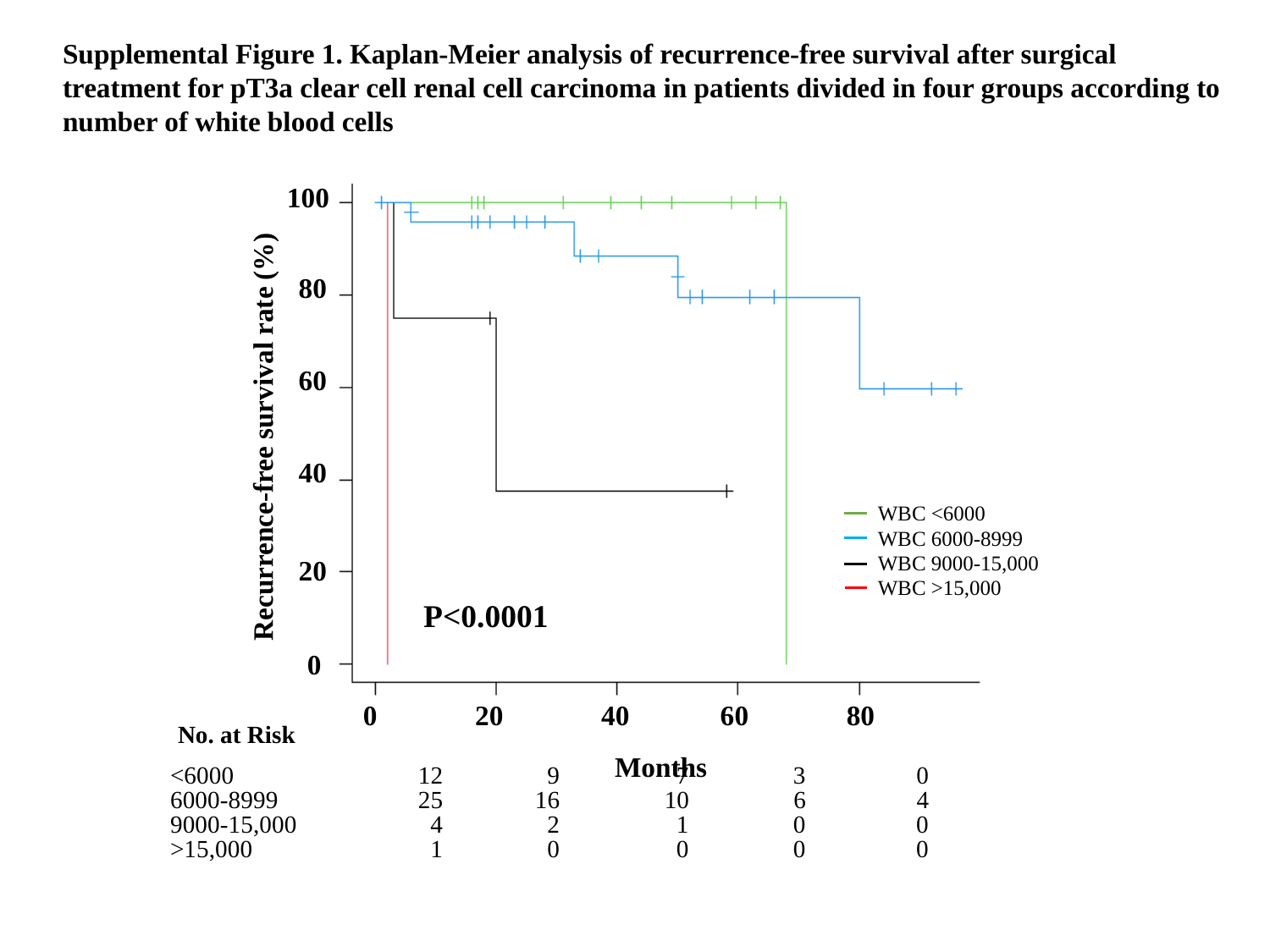

Supplemental Figure 1. Kaplan-Meier analysis of recurrence-free survival after surgical treatment for pT3a clear cell renal cell carcinoma in patients divided in four groups according to number of white blood cells
100
80
60
40
20
0
Recurrence-free survival rate (%)
WBC <6000
WBC 6000-8999
WBC 9000-15,000
WBC >15,000
P<0.0001
0 20 40 60 80
 Months
No. at Risk
 <6000		12 9 7 3 0
 6000-8999	 	25 16 10 6 4
 9000-15,000 	 4 2 1 0 0
 >15,000		 1 0 0 0 0
